# Coronal Microleakage in Root Canals Obturated with Lateral Compaction, Warm Vertical Compaction and Guttaflow System

**Published:** 2010-05-20

**Authors:** Mohsen Aminsobhani, Abdollah Ghorbanzadeh, Behnam Bolhari, Noushin Shokouhinejad, Sholeh Ghabraei, Hadi Assadian, Marzieh Aligholi

**Affiliations:** 1. Department of Endodontics, Dental School/Dental Research Center, Tehran University of Medical Sciences, and member of Iranian Center for Endodontic Research, Tehran, Iran.; 2. Department of Endodontics, Dental School/Dental Research Center, Tehran University of Medical Sciences, Tehran, Iran.; 3. Department of Endodontics, Dental School, Jondi Shapour University of Medical Sciences, Ahvaz, Iran.; 4. Department of Microbiology, School of Medicine, Tehran University of Medical Sciences, Tehran, Iran.

**Keywords:** Dental Leakage, GuttaFlow, Gutta-Percha, Microleakage, Root Canal Obturation

## Abstract

**INTRODUCTION:**

Root canal obturation seals the root canal system to prevent re-entry and/or growth of microorganisms. The provision of an appropriate restoration to coronally seal the access cavity affects the success of endodontic treatment. The purpose of this study was to evaluate the coronal microbial leakage in root canals that were either filled by lateral compaction, GuttaFlow or warm vertical compaction.

**MATERIALS AND METHODS:**

In this ex vivo study, 80 single-rooted human extracted teeth were randomly divided into three experimental groups (n=20) and two positive and negative control groups (n=10). The teeth in experimental groups were obturated with cold lateral compaction, GuttaFlow system or warm vertical compaction techniques. After sterilization of the whole system with gamma-ray, saliva leakage was tested using a split-chamber model. Specimens were monitored every 24 hours for 30 days. The data were analyzed using log-rank and Kaplan-Meier survival analysis tests.

**RESULTS:**

There were no significant differences in impeding saliva leakage between the three experimental groups (P>0.05).

**CONCLUSION:**

Under the conditions of this ex vivo study, it can be concluded that the sealing ability of cold lateral compaction, warm vertical compaction and GuttaFlow system was comparable.

## INTRODUCTION

The purpose of root canal obturation, which is an essential part of root canal treatment, is to prevent the re-entry and growth of microorganisms and to trap remnant traces of pathogens inside the root canal system [1]. The penetration of bacteria and their by-products from the oral cavity into the obturated root canals jeopardizes the endodontic treatment success. Therefore, evaluating the quality of root canal obturation as the final stage of root canal treatment is essential [2]. Moreover, well designed studies have shown that an appropriate coronal seal as well as a complete apical seal greatly enhances the success of endodontic treatments [3]. Until now, different methods have been introduced for root canal obturation; an appropriate method should be able to prevent the re-entry of microorganisms into the root canals. Various root canal obturation methods have shown different degrees of sealability; this difference is due to the materials’ adaptation with canal walls and their penetration into lateral canals and dentinal tubules [4][5]. The sealing ability of lateral compaction, vertical compaction, and the other obturation methods has been evaluated [4][5]. Warm sectional vertical compaction obturation achieved a superior seal when compared with lateral compaction obturation methods. However, other studies were not able to confirm this difference [6][7]. An interesting in vitro fluid filtration study showed that after 2 weeks, apical sealing efficiency of two warm vertical compaction techniques were inferior to the cold lateral compaction [8].

One of the most reliable methods for evaluating this leakage is utilizing microorganisms that exist in saliva [2].

The present study was conducted to evaluate the microbial leakage along the root canals filled by lateral compaction, GuttaFlow and warm vertical compaction techniques.

## MATERIALS AND METHODS

In this ex vivo study, 80 straight single-rooted human extracted teeth devoid of cracks (viewed under three magnifications) were selected and disinfected. All teeth were decoronated to obtain 13-mm long specimens. The working length was established visually by subtracting 1 mm from the length of a K-file size #15 (Dentsply, Maillefer, Ballaigues, Switzerland) placed at the apical foramen. Root canals were instrumented with the crown down method using rotary FlexMaster nickel-titanium files (VDW, Munich, Germany) up to the apical file size #40, with 0/06 taper. During canal preparation, 1 mL sodium hypochlorite 2.5% was used for irrigation. Towards the end of canal preparation, root canals were irrigated with 1 mL EDTA 17% (MD-Cleanser™, Meta Biomed Co. Ltd., Cheongju City, Chungbuk, Korea) followed by 5 mL sodium hypochlorite 2.5% to remove the smear layer. The root canals were finally flushed with 5 mL of normal saline and dried with paper points (Meta Biomed Co., Ltd., Cheougja City, Chungbuk, Korea). The teeth were randomly divided into three experimental groups of 20 each, and two positive and negative control groups (n=10). The teeth in experimental groups were obturated as follows: group 1: gutta-percha/AH26 sealer using lateral compaction technique; group 2: GuttaFlow system; group 3: gutta-percha/AH26 sealer using warm vertical compaction technique and thermoplastic gutta-percha injection.

In the lateral compaction method, the root canals were obturated with master cone size #40, 0.02 tapered gutta-percha (Meta Biomed Co., LTd., Cheongju City, Chungbuk, Korea) and size #30, 0.02 tapered gutta-percha as accessory cones and with AH26 sealer (De Trey Dentsply, Switzerland) by using a #35 stainless steel spreaders (Mani Inc., Tochigi, Japan). In the second group, root canals were obturated with GuttaFlow system that consists of a polydimethylsiloxane-based root canal ﬁlling material (GuttaFlow; Coltene/Whaledent GmbH + Co.KG, Langenau, Germany). The third group used hand pluggers (Machtou's heat-carrier pluggers; Dentsply-Maillefer, Ballaigues, Switzerland) with size #40, 0.06 tapered gutta-percha points (Meta Biomed Co., LTd., Cheongju City, Chungbuk, Korea) for the apical two-thirds. For obturation of the coronal 2/3 of the root canals in back pack procedure, an injection device for injecting the gutta-percha, Cordless Gutta-percha obturation Gun (E and Q Master, Meta Biomed Co., Ltd, Cheongju city, Chungbuk, Korea), was used. The quality of root canal obturation of all samples was confirmed with a digital radiography taken by Kodak device (Kodak RVG 5100, Eastman Kodak Company, Trophy Radiologie S.A, Marne-la-Valee, France).

All teeth were kept at 37˚C and 100% humidity for 7 days. Except for the apical 3 mm, all other segments of teeth in all groups received two layers of nail varnish (Arcancil, Paris, France). The roots in negative control group were coated completely with two layers of nail varnish. A split-chamber model was used for bacterial leakage evaluation. The taper end of the 2-mL plastic Eppendorf tube (Elkay, Shrewbury, MA, USA) was cut and each root was placed into the tube. Roots were positioned inside the tube so that their apical end was removed from the cut-end part of the Eppendorf tube. The junction between the plastic tube and the root was sealed with sticky wax. The apparatus composed of teeth and plastic tubes were sterilized by exposure to 40 kGray Gamma irradiation. The specimens were incubated at 37˚C for 3 days to confirm system.

Subsequently, under the sterilized conditions (i.e. under the hood), the Eppendorf tube samples were placed in a glass tube containing Brain Heart Infusion (BHI) broth (Merck Eurolab, Darmstadt, Germany), so that at least 2 mm of the root apex was in BHI. The junction between the Eppendorf and the glass tube was sealed tightly with sticky wax. Samples were kept for 3 days under 37˚C in an incubator to confirm sterility of the procedure. If turbidity was observed in the BHI, the sample was again sterilized. Once the samples were deemed sterile, the upper chamber of the split-model was filled with 2 mL of human saliva. The saliva was changed every 3 days. Human saliva was selected from a volunteer that had not observed oral hygiene for at least 12 hours before saliva collection [9]. The samples were incubated at 37˚C and evaluated daily for the presence of turbidity in the lower chamber of the system. The data were analyzed by Survival Analysis and Log-Rank tests.

## RESULTS

**Figure 1:**
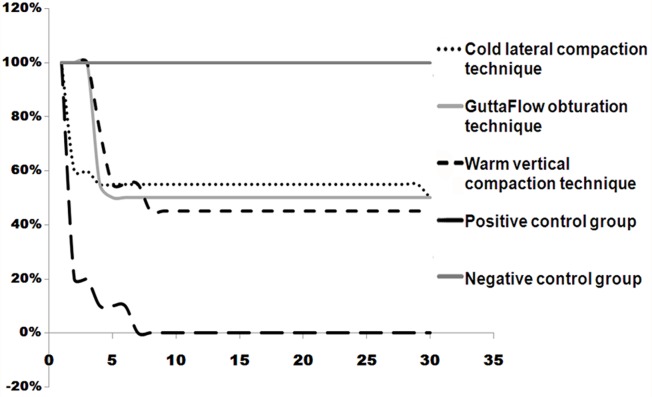
Survival curve of non-leaked samples (Percent of non-leaked samples in 30 days period of experiment).

All samples in the positive control group showed leakage, whereas the negative control group showed zero leakage during the 30-day trial. The number and percentage of samples which leaked in each group and the mean ± SD leakage time for are shown in Table 1. The survival curve of non-leaked samples (percentage of non-leaked samples in 30 days) is shown in Figure 1.

**Table 1:** Number and (%) of positive leakage samples and the average time required for leakage to occur at the end of a 30-day period

**Group**	**Number (%) of positive leakage samples**	**Time needed for leakage in days (Mean ± SD)**
**Lateral compaction**	10 (50%)	16.5 ± 3.26
**GuttaFlow system**	10 (50%)	16.05 ± 2.90
**Warm Vertical compaction**	11 (55%)	15.30 ± 2.78

Positive control samples showed turbidity in lower chambers during the first 7 days. The appearance of turbidity first occurred on the 2nd day in group 1 and positive control group; for groups 2 and 3 it occurred on the 4th day.

Survival analysis and log-rank test showed no significant difference between the groups at the end of the 30-day study (P>0.05).

## Discussion

The efficacy of root canal obturations’ sealibility greatly affects endodontic treatment outcome. The differences in study methods have however, resulted in various results and even controversies. Methods that are usually used in these kinds of studies are: 1- dye penetration [7], 2- electrochemical leakage tests [10][11], 3- fluid filtration [8][12], 4- bacterial leakage [13], and 5- salivary leakage (polymicrobial leakage) [14].

This study used a modification of the salivary leakage system with two chambers for evaluating microleakage (designed by Torabinejad et al.) [15]. Human saliva was chosen to try to simulate the clinical condition. Despite some controversies, most studies recommend smear layer removal to assist the obturating material’s adaptability to the root canals [16][17]. Therefore, this study removed the smear layer with NaOCl 2.5% and EDTA 17% irrigants.

Obturation was performed with AH26 (epoxy-resin-based sealer) in all groups. AH26 has low contraction and solubility in comparison with ZOE-based and calcium hydroxide root canal sealers resulting in lower leakage [18][19][20].

According to Pommel and Camps, samples should be follow up for a period of 30 days [21], as in this study. The results of longer evaluation times can result in more precise data; however, foul odor of remaining saliva in the coronal chambers prevent longer evaluations. The findings of our study showed that the sealing ability of lateral compaction, vertical compaction and GuttaFlow system were not significantly different. Our results are in accordance with Chohayeb who used the dye leakage method [22]. Monticelli et al. compared warm vertical compaction, GuttaFlow, single cone Active GP and showed that the warm vertical compaction method had superior seal compared with the other two obturation methods [13]. This difference may be attributed to the difference in sealer thicknesses in these three methods of obturation [14]; vertical compaction method has the lowest thickness. In Tay et al.’s study, the existence of irregularities in canals was regarded as an important factor in the microleakage of GuttaFlow technique [23]. According to Elayouti et al., voids in GuttaFlow and between sealer and canal walls can lead to increase in coronal microleakage [24]. Other studies have shown that lateral compaction obturations had greater microleakage due to the presence of voids and non homogenized obturations, they had the least amount of gutta-percha mass volume, and therefore were unable to penetrate into canal irregularities [25]. However, the results of our study showed no significant differences between the sealing ability of lateral compaction and other two obturation methods: warm vertical compaction and GuttaFlow system.

Similarly, Brackett et al. did not find any significant difference in the sealing capacity between GuttaFlow, warm vertical compaction and thermoplastic gutta-percha with AH plus sealer using fluid filtration method for evaluating the microleakage [12]. Furthermore, another study that investigated sealing ability with three different sealers and the fluid filtration technique did not observe a significant difference between thermafill and different lateral compaction methods [26].

The evaluation of sealing ability of gutta-percha obturation using saliva leakage method is based on the salivary hydrolytic enzymes ability to break the seal. These microbial productions destroy and decompose gutta-percha resulting in the loss of the adaptation of gutta-percha to canal walls, thereby reducing the coronal seal. In Maniglia-Ferreira et al.’s study, decomposition and destruction of polyisoprene (the main substance of gutta-percha) produced high amounts of carboxyl and hydroxyl radicals during thermo mechanical compaction and thermoplastic techniques [27]. These resulted in molecular weight reduction and a decrease in the stability and sealing ability of the obturating substances, thereby increasing coronal microleakage [27]. Similarly, in our study, a large amount of samples leaked within 15-16 days, possibly due to this effect.

Therefore, application of coronal temporization and restoration is necessary to prevent the re-infection of the root canal space post-obturation [3].

## CONCLUSION

Under the conditions of this ex vivo study, no statistical difference was found between lateral compaction, GuttaFlow, and warm vertical compaction sealing ability. However, further clinical investigations should be performed for definitive conclusions. Moreover, it would be reasonable to recommend that the crowns of the endodontically treated teeth be adequately restored as soon as possible.

## References

[1] Gilbert SD, Witherspoon DE, Berry CW (2001). Coronal leakage following three obturation techniques. Int Endod J.

[2] Monticelli F, Sword J, Martin RL, Schuster GS, Weller RN, Ferrari M, Pashley DH, Tay FR (2007). Sealing properties of two contemporary single-cone obturation systems. Int Endod J.

[3] Yamauchi S, Shipper G, Buttke T, Yamauchi M, Trope M (2006). Effect of orifice plugs on periapical inflammation in dogs. J Endod.

[4] Gopikrishna V, Parameswaren A (2006). Coronal sealing ability of three sectional obturation techniques--SimpliFill, Thermafil and warm vertical compaction--compared with cold lateral condensation and post space preparation. Aust Endod J.

[5] Chailertvanitkul P, Saunders WP, Saunders EM, MacKenzie D (1997). An evaluation of microbial coronal leakage in the restored pulp chamber of root-canal treated multirooted teeth. Int Endod J.

[6] Pérez Heredia M, Clavero González J, Ferrer Luque CM, González Rodríguez MP (2007). Apical seal comparison of low-temperature thermoplasticized gutta-percha technique and lateral condensation with two different master cones. Med Oral Patol Oral Cir Bucal.

[7] Namazikhah S, Shirani R, Mohseni A, Farsio F (2000). Dye leakage study: comparing conventional and new techniques. J Calif Dent Assoc.

[8] Yilmaz Z, Deniz D, Ozcelik B, Sahin C, Cimilli H, Cehreli ZC, Kartal N (2009). Sealing efficiency of BeeFill 2in1 and System B/Obtura II versus single-cone and cold lateral compaction techniques. Oral Surg Oral Med Oral Pathol Oral Radiol Endod.

[9] Gomes BP, Sato E, Ferraz CC, Teixeira FB, Zaia AA, Souza-Filho FJ (2003). Evaluation of time required for recontamination of coronally sealed canals medicated with calcium hydroxide and chlorhexidine. Int Endod J.

[10] Amditis C, Bryant RW, Blackler SM (1993). The assessment of apical leakage of root-filled teeth by the electrochemical technique. Aust Dent J.

[11] Karagenç B, Gençoglu N, Ersoy M, Cansever G, Külekçi G (2006). A comparison of four different microleakage tests for assessment of leakage of root canal fillings. Oral Surg Oral Med Oral Pathol Oral Radiol Endod.

[12] Brackett MG, Martin R, Sword J, Oxford C, Rueggeberg FA, Tay FR, Pashley DH (2006). Comparison of seal after obturation techniques using a polydimethylsiloxane-based root canal sealer. J Endod.

[13] Monticelli F, Sadek FT, Schuster GS, Volkmann KR, Looney SW, Ferrari M, Toledano M, Pashley DH, Tay FR (2007). Efficacy of two contemporary single-cone filling techniques in preventing bacterial leakage. J Endod.

[14] De-Deus G, Coutinho-Filho T, Reis C, Murad C, Paciornik S (2006). Polymicrobial leakage of four root canal sealers at two different thicknesses. J Endod.

[15] Torabinejad M, Ung B, Kettering JD (1990). In vitro bacterial penetration of coronally unsealed endodontically treated teeth. J Endod.

[16] Clark-Holke D, Drake D, Walton R, Rivera E, Guthmiller JM (2003). Bacterial penetration through canals of endodontically treated teeth in the presence or absence of the smear layer. J Dent.

[17] Cobankara FK, Adanr N, Belli S (2004). Evaluation of the influence of smear layer on the apical and coronal sealing ability of two sealers. J Endod.

[18] Sevimay S, Kalayci A (2005). Evaluation of apical sealing ability and adaptation to dentine of two resin-based sealers. J Oral Rehabil.

[19] Limkangwalmongkol S, Abbott PV, Sandler AB (1992). Apical dye penetration with four root canal sealers and gutta-percha using longitudinal sectioning. J Endod.

[20] De Almeida WA, Leonardo MR, Tanomaru Filho M, Silva LA (2000). Evaluation of apical sealing of three endodontic sealers. Int Endod J.

[21] Pommel L, Camps J (2001). In vitro apical leakage of system B compared with other filling techniques. J Endod.

[22] Chohayeb AA (1992). Comparison of conventional root canal obturation techniques with Thermafil obturators. J Endod.

[23] Tay FR, Loushine RJ, Lambrechts P, Weller RN, Pashley DH (2005). Geometric factors affecting dentin bonding in root canals: a theoretical modeling approach. J Endod.

[24] Elayouti A, Achleithner C, Löst C, Weiger R (2005). Homogeneity and adaptation of a new gutta-percha paste to root canal walls. J Endod.

[25] Kandaswamy D, VenkateshBabu N, Reddy GK, Hannah R, Arathi G, Roohi R (2009). Comparison of laterally condensed, vertically compacted thermoplasticized, cold free-flow GP obturations - A volumetric analysis using spiral CT. J Conserv Dent.

[26] Schäfer E, Olthoff G (2002). Effect of three different sealers on the sealing ability of both thermafil obturators and cold laterally compacted Gutta-Percha. J Endod.

[27] Maniglia-Ferreira C, Bönecker G, Silva JB Jr, de Paula RC, Feitosa JP, Souza-Filho FJ (2008). Degradation of trans-polyisoprene after root filling with thermoplasticized techniques. Int Endod J.

